# Development and Optimization of an ELISA to Quantitate C3(H_*2*_O) as a Marker of Human Disease

**DOI:** 10.3389/fimmu.2019.00703

**Published:** 2019-04-04

**Authors:** Michelle Elvington, M. Kathryn Liszewski, Alexis R. Liszewski, Hrishikesh S. Kulkarni, Ramsey R. Hachem, Thalachallour Mohanakumar, Alfred H. J. Kim, John P. Atkinson

**Affiliations:** ^1^Division of Rheumatology, Department of Medicine, Washington University School of Medicine, St. Louis, MO, United States; ^2^Division of Pulmonary and Critical Care Medicine, Department of Medicine, Washington University School of Medicine, St. Louis, MO, United States; ^3^Norton Thoracic Institute, St. Joseph's Hospital and Medical Center, Phoenix, AZ, United States

**Keywords:** complement, C3, human, autoimmunity, ELISA, C3(H2O), biomarker

## Abstract

Discovery of a C3(H_2_O) uptake pathway has led to renewed interest in this alternative pathway triggering form of C3 in human biospecimens. Previously, a quantifiable method to measure C3(H_2_O), not confounded by other complement activation products, was unavailable. Herein, we describe a sensitive and specific ELISA for C3(H_2_O). We initially utilized this assay to determine baseline C3(H_2_O) levels in healthy human fluids and to define optimal sample storage and handling conditions. We detected ~500 ng/ml of C3(H_2_O) in fresh serum and plasma, a value substantially lower than what was predicted based on previous studies with purified C3 preparations. After a single freeze-thaw cycle, the C3(H_2_O) concentration increased 3- to 4-fold (~2,000 ng/ml). Subsequent freeze-thaw cycles had a lesser impact on C3(H_2_O) generation. Further, we found that storage of human sera or plasma samples at 4°C for up to 22 h did not generate additional C3(H_2_O). To determine the potential use of C3(H_2_O) as a biomarker, we evaluated specimens from patients with inflammatory-driven diseases. C3(H_2_O) concentrations were moderately increased (1.5- to 2-fold) at baseline in sera from active systemic lupus erythematosus (SLE) and rheumatoid arthritis (RA) patients compared to healthy controls. In addition, upon challenge with multiple freeze-thaw cycles or incubation at 22 or 37°C, C3(H_2_O) generation was significantly enhanced in SLE and RA patients' sera. In bronchoalveolar lavage fluid from lung-transplant recipients, we noted a substantial increase in C3(H_2_O) within 3 months of acute antibody-mediated rejection. In conclusion, we have established an ELISA for assessing C3(H_2_O) as a diagnostic and prognostic biomarker in human diseases.

## Introduction

The complement system is a well-established component of innate immunity and an instructor of adaptive immunity. An important function of the complement system is to identify and opsonize targets, including microbes, immune complexes, apoptotic cells and necrotic tissue, to facilitate their “safe” removal. Complement activation occurs primarily through three pathways, the classical (CP), lectin (LP), and alternative (AP). While the CP and LP have specific triggers (antibodies and lectins, respectively), the AP is thought to be independently engaged by the spontaneous activation of C3 to a form with a cleaved thioester bond (1, 2). The prevailing hypothesis is that this occurs by the spontaneous, low-level hydrolysis of the internal thioester bond in native C3 in the fluid phase (3–5). Within microseconds this form of C3 becomes covalently bound to a target (such as a microbe or cell) or interacts with water to form C3(H_2_O) (4). C3(H_2_O) can then bind factor B (FB) after which factor D cleaves FB to Bb and Ba. The C3 converting enzyme or convertase, C3(H_2_O)Bb, then generates C3b from C3 to initiate AP activity on a nearby target (4, 6). To regulate this system, factor H (FH) binds C3(H_2_O) and the serine protease factor I (FI) can then cleave it to a form that cannot amplify the cascade (2, 7, 8). Since C3(H_2_O) may be a trigger for complement activation at sites of cellular injury, degeneration or autoimmune attack, a simple, quantitative and specific assay for this form of C3 could provide insights into how the complement system functions in human disease.

Undesirable and excessive activation of C3 is a feature of multiple autoimmune- and inflammatory-driven diseases (9, 10). Interest in C3(H_2_O) in disease processes has recently been renewed due to the discovery of the intracellular complement system (ICS) whose dysfunction is increasingly associated with impaired cellular metabolism, debris clearance and autoimmunity (11–19). In a follow-up report to the identification of the ICS, we made an additional key observation of a process in which C3(H_2_O) is loaded into the cell interior where it can be stored or recycled back out of the cell (20). Importantly, this represents an exogenous shuttling pathway in which the transported C3(H_2_O) could modulate immune cell functions, such as IL-6 secretion (20). Thus, C3(H_2_O) generation is functionally relevant as a trigger for the AP in the extracellular space (3, 4) and, once generated, it can also be loaded into cells to play a role in the ICS (20).

We hypothesize that C3(H_2_O) is a major player in cellular homeostasis whose upregulation may serve as a biomarker of inflammatory processes. Prior ELISA assessments of C3(H_2_O) were confounded by reactivity to additional fragments, especially C3a (21, 22). Herein, we describe the development of an ELISA that is specific for C3(H_2_O). The selectivity is based, first, on the capture of the conformationally altered structure of C3(H_2_O) and, second, by detection of the C3a domain. Using this ELISA, we have initially characterized the specificity of this assay, optimized specimen handling and quantified levels in human serum and plasma from healthy donors. Additionally, we have determined levels of C3(H_2_O) that are generated under various potential modifiers and in disease situations that will provide guidance as to how human samples should be handled and stored in clinical and laboratory settings.

## Materials and Methods

### Antibodies and Proteins

Purified C3, C3b, C3a, iC3b, FH, and FI were obtained from Complement Technologies. Complement receptor 1 (CR1; CD35) was from Avant Immunotherapeutics. The mouse anti-C3b/iC3b [clone 3E7, (23) and specificity for iC3b confirmed in (24)] was a generous gift from Dr. Ronald Taylor (University of Virginia Health Sciences Center) and was also purchased from MilliporeSigma. Rabbit anti-human C3a (A218) and goat anti-human C3 (A213) polyclonal antibodies (pAbs) were also purchased from Complement Technologies. Chicken anti-human C3 (GW20073F) pAb was obtained from MilliporeSigma.

### Sample Processing

Whole blood was collected by venipuncture in commercial blood collection tubes (BD Biosciences Vacutainer). Serum samples were allowed to clot for 30 min at RT prior to centrifugation at 2,000 x g for 5 min. Plasma samples were centrifuged at RT immediately at 2,000 x g for 10 min. Samples were either evaluated immediately (fresh) or stored in aliquots at −80°C until use. Bronchoalveolar lavage (BAL) fluid was obtained from lung transplant recipients by instilling phosphate-buffered saline into the airway via a bronchoscope followed by aspiration. The fluid was transported at 4°C after which cells were separated by centrifugation at 500 x g for 10 min at 4°C. BAL samples were stored at −80°C until use.

### C3 Handling and Preparation

To prepare a C3(H_2_O) analog known as C3(MA), we treated purified C3 with methylamine to hydrolyze the thioester bond (3, 20). Briefly, purified C3 was incubated with 0.4 M methylamine hydrochloride (Sigma Aldrich) and 0.1 M Tris pH 8.0 for 1 h at 37°C. Another analog, C3(KBr), was generated by treatment of purified C3 with 2 M potassium bromide for 3 h at 37°C (25). Freeze/thaw cycles of serum and plasma were performed by rapidly thawing 100 μl samples at 37°C in a water bath. Immediately after thawing, the C3 was placed on ice and returned to the −80°C freezer for 15 min. Freeze/thaw cycles of purified C3 were performed by thawing 100 μl aliquots of 1 mg/ml C3 at 22°C, followed by freezing at −80°C.

### ELISA Protocol

Maxisorp 96-well ELISA plates were coated with 100 μl/well of 2 μg/ml 3E7 [anti-C3b/iC3b antibody characterized in (23)] diluted in PBS overnight at 4°C. The plates were washed 3x with PBS containing 0.05% Tween-20 (PBST) and blocked for 1 h at RT with PBST + 4% BSA (blocking buffer). Samples and standards were diluted in blocking buffer, added to plate and allowed to incubate at RT for 1 h. Rabbit anti-C3a was diluted 1:4000 in blocking buffer and incubated for 1 h at RT. HRP-conjugated donkey anti-rabbit IgG secondary was diluted 1:5000 in the same buffer and then incubated for 1 h at RT. Following washing, tetramethylbenzidine (TMB) substrate was added. Following a 15 min incubation, the reaction was stopped with 1 M phosphoric acid and absorbance read at 450 nM on a uQuant plate reader (BioTek U.S., Winooski, VT).

### Cofactor Assays

Cofactor assays were performed as previously described (26). Briefly, *in vitro* cleavage of C3b and C3(MA) by FI in the presence of the cofactor FH or sCR1 was performed by incubating 40 ng of C3b or C3(MA) with 500 ng FH or sCR1 and 50 ng FI at 37°C for 30 min.

### Western Blotting

Protein samples were electrophoresed under reducing conditions in 4–20% SDS-PAG, transferred to nitrocellulose, and then probed with rabbit anti-human C3a pAb or goat anti-human C3 pAb (Complement Technologies). This was followed by appropriate HRP-conjugated secondary Abs. Blots were visualized on a ChemiDoc XRS Imager (Bio-Rad).

### Study Approval

Blood and BAL fluid were collected from healthy donors and patients after written informed consent was obtained according to the guidelines of the Washington University School of Medicine Human Studies Committee. The SLE patient samples are part of the cohort described in Kim et al. (27).

### Statistics

All data were subjected to statistical analysis using Prism software version 7 (GraphPad). Comparisons between two groups were performed by 2-tailed *t*-test (parametric) or paired *t-*test (non-parametric). Comparisons between multiple groups were performed using a 1-way ANOVA with Bonferroni's multiple comparison test. A *P* < 0.05 was considered significant.

## Results

### Development of an ELISA Specific for C3(H_2_O)

**Figure 1:**
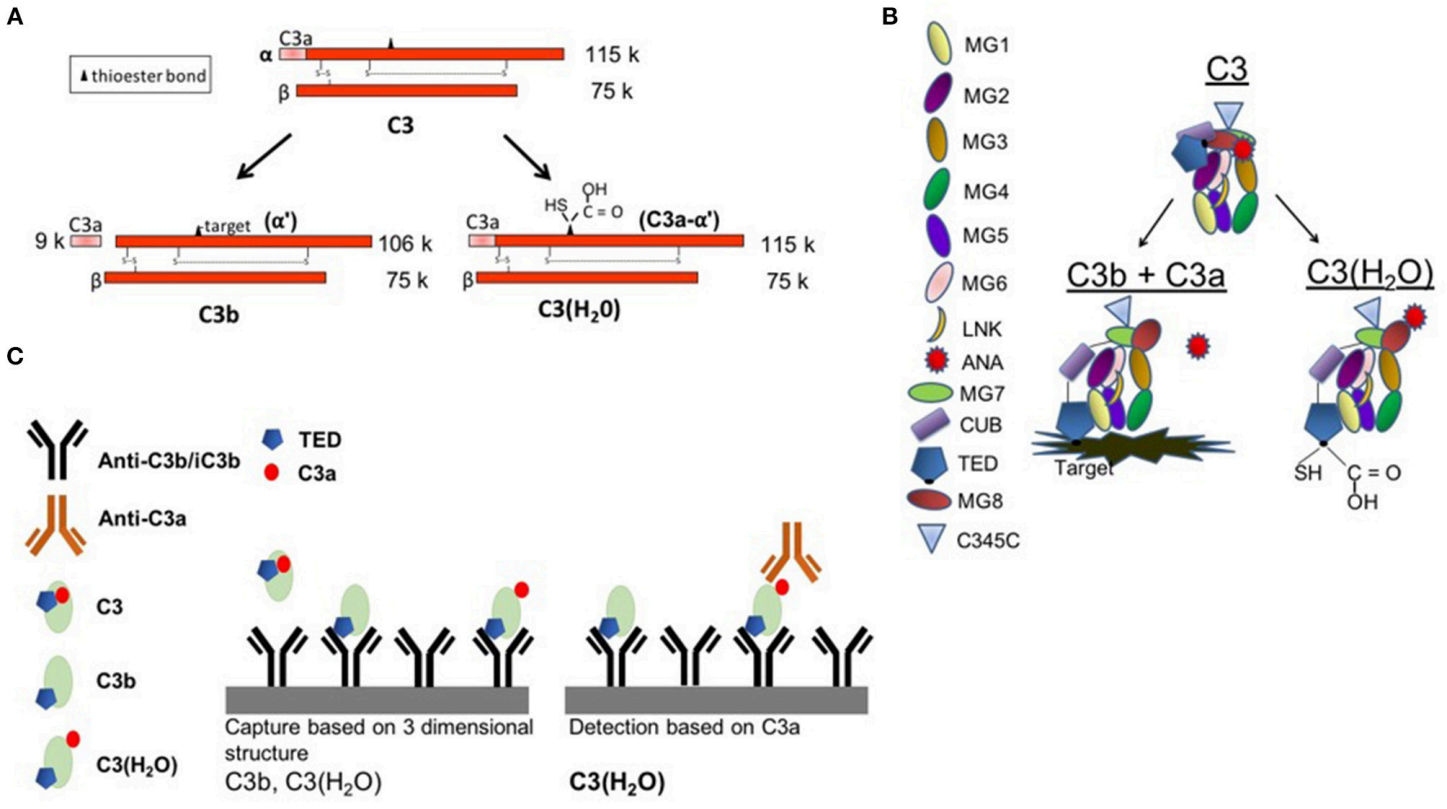
C3(H_2_O) ELISA set-up. **(A)** Schematic depicting C3, C3b, and C3(H_2_O). **(B)** Diagram showing protein domains and the conformational rearrangement following C3 activation to either C3b (with liberation of C3a) or C3(H_2_O) (with a hydrolyzed thioester and retention of C3a). Note the movement of the ANA domain (red) and the TED domain (dark blue). Diagram adapted from Janssen et al. (34, 35) and Gros et al. (36). **(C)** C3b and C3(H_2_O) are recognized by the capture antibody (3E7, mouse anti-C3b/iC3b). C3 is not captured. C3(H_2_O) is recognized based on the presence of the C3a (ANA) domain in the detection step. MG1-8, macroglobulin domains 1-8; LNK, linker domain; ANA, anaphylatoxin domain; α′NT, n-terminus of the α-chain; CUB, complement C1r/C1s, UEGF, BMP1; TED, thioester-containing domain; C345C, netrin (NTR) domain.

To determine if C3(H_2_O) might serve as a disease biomarker for conditions associated with complement activation as well as to further evaluate the sample handling issues, we set out to develop an ELISA specific for C3(H_2_O) that lacks cross-reactivity with C3, C3b or C3b fragments (Figure 1A). Antigenic assays to quantitate C3 typically recognize both C3 and C3(H_2_O) (Figure 1A). Importantly, the hydrolysis of the internal thioester bond is accompanied by a substantial conformational change in C3(H_2_O) such that it closely mimics the three-dimensional structure of C3b (28–35) (Figure 1B). Accordingly, we designed a C3(H_2_O)-specific ELISA in which the capture antibody (Ab) recognizes a conformationally-specific epitope in C3(H_2_O) and C3b, while the other Ab exclusively detects C3a (Figure 1C). Thus, C3b and C3(H_2_O) were separated from C3 during the capture step and C3(H_2_O) was distinguished from C3b by the detection Ab being specific for the C3a domain (Figure 1C).

**Figure 2:**
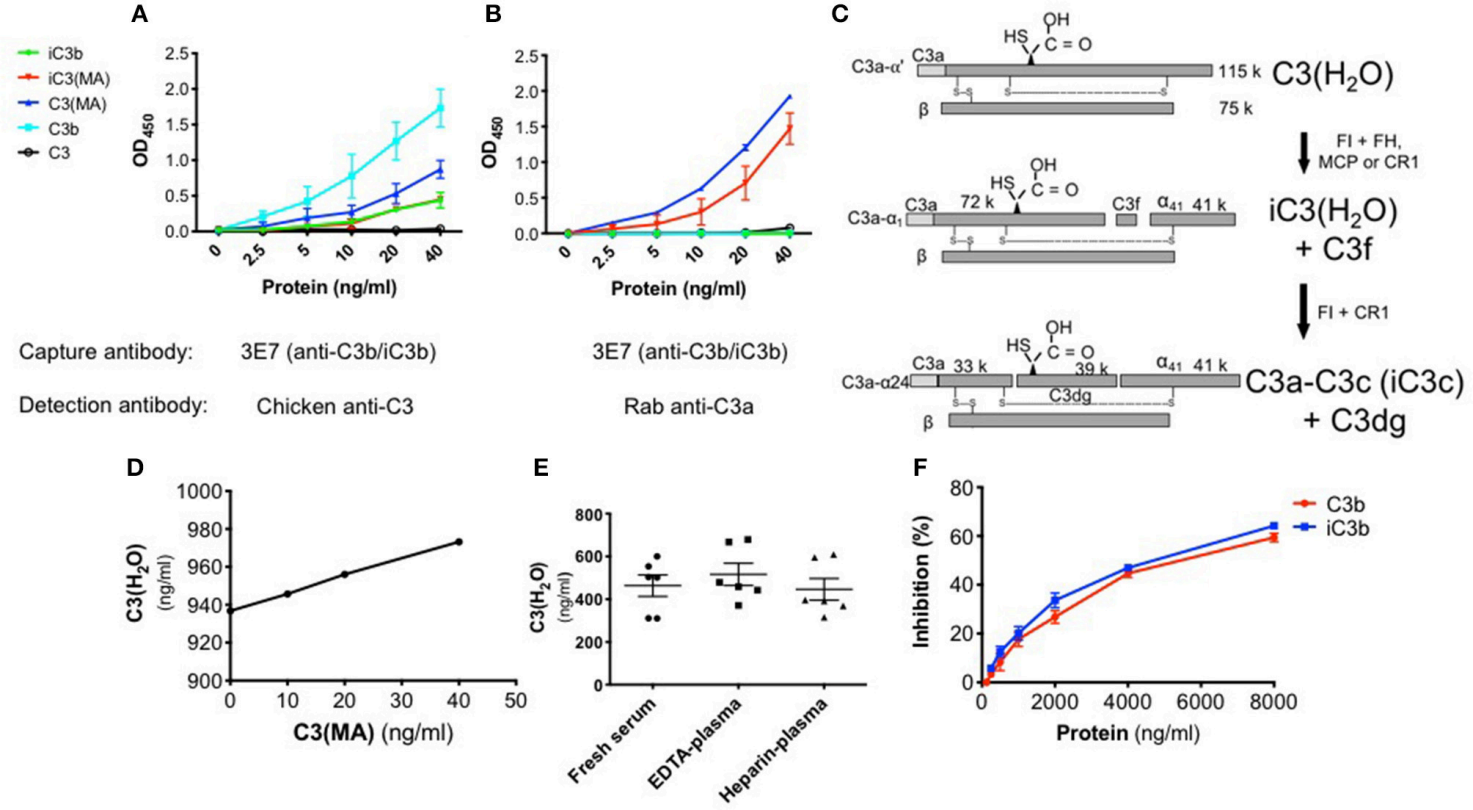
Specificity of the C3(H_2_O) ELISA. **(A)** Binding comparison of proteins using anti-C3 pAb for detection**. (B)** Specificity of ELISA employing anti-C3a pAb. C3(MA) and iC3(MA) are detected. **(C)** Schematic depicting the cleavage and inactivation of C3(H_2_O) by cofactor proteins and the serine protease FI. Adapted from Elvington et al. (20). **(D)** Increasing concentrations of C3(MA) were spiked into 1:100 NHS which had a baseline C3(H_2_O) level of 938 ng/ml and protein recovery of the C3(MA) over baseline was assessed by ELISA; *n* = 2, representative of 2 independent experiments. **(E)** C3(H_2_O) was measured in human serum and plasma from healthy adults; *n* = 3 donors (in duplicate, intra-assay precision; %CV 4.97). **(F)** Increasing quantity of purified C3b (red) or iC3b (blue) was incubated with 40 ng/ml C3(MA) to assess if they compete for binding; *n* = 3.

To confirm the specificity of the C3(H_2_O) ELISA, we utilized purified proteins to determine the selectivity of the capture and detection Abs. A polyclonal anti-C3 detection Ab was employed to evaluate the selectivity of the capture Ab. We confirmed that in our assay the anti-C3b/iC3b capture Ab binds C3 methylamine [C3(MA), a C3(H_2_O) analog] and C3b/iC3b but not native C3 (Figure 2A) (23). The small amount of signal from purified C3 (open circle, Figures 2A,B) was likely from the trace quantity of C3(H_2_O) in the preparation and not from captured C3 (20, 23).

Since C3(H_2_O) retains the C3a fragment which is lacking in C3b/iC3b, we distinguished between these two proteins during the detection step utilizing an anti-C3a pAb. The specificity of the detection pAb for C3a-containing proteins was confirmed as C3b was not identified (Figure 2B), although, as expected, C3b was confirmed to be captured using the anti-C3 pAb for detection (Figure 2A). Purified C3(MA) was used to prepare a standard curve and establish that the sensitivity of the assay ranges from 1.25 to 40 ng/ml (Figure 2B). C3(MA) was selected as a surrogate for C3(H_2_O) for the standard curve because it has been established that it is conformationally and functionally similar to C3(H_2_O) (20, 25, 37). First, the C3(MA) elution profile corresponds to C3(H_2_O) in studies demonstrating that C3 and C3(H_2_O) can be separated by cation exchange chromatography (37). Second, experiments in which C3 and C3(H_2_O) were purified from human serum demonstrated that C3(MA) behaves similarly to C3(H_2_O) in cellular uptake (20). Further, treatment of C3 with KBr has been reported to result in a conformational species corresponding to C3(H_2_O) (25) and we have used C3(KBr) in our ELISA with analogous results to C3(MA) (Figure S1A).

Certain breakdown fragments of C3(H_2_O) retain C3a (Figure 2C). Consequently, if evaluating physiologic samples such as human sera, there is a possibility that the C3(H_2_O) levels will be falsely elevated due to the presence of these fragments. Thus, we evaluated whether the ELISA could also detect these downstream fragments. We generated iC3(MA) and C3a-C3c (see Methods) and confirmed conversion of the C3(MA) to iC3(MA) and C3a-C3c by Western blot (Figures 2C; S1B). Using these purified proteins in our ELISA, we demonstrated that iC3(MA) is detected (Figure 2B); however, following cleavage to C3a-C3c, binding is lost (Figure S2). Additionally, we confirmed that C3a and C3c are not detected in the ELISA (Figure S2). This indicates that only C3(H_2_O) and iC3(H_2_O) will be detected in human samples and that downstream degradation fragments, even those containing C3a, will not alter C3(H_2_O) measurements.

### ELISA Validation and Optimization in Human Biospecimens

Matrix effects can occur when target antigen interacts with other components in serum. To validate the use of the ELISA and to ensure we could overcome potential inhibitory effects of serum (matrix effects), we spiked in increasing concentrations of C3(MA) to undiluted or 1:100 normal human serum (NHS) and monitored recovery of the protein. In undiluted NHS, we did not detect C3(H_2_O). We also did not detect C3(MA) that is spiked into the sample, indicating that undiluted NHS is inhibitory in the ELISA; i.e., it causes a matrix effect. In contrast, at a 1:100 dilution of NHS, we fully detected the spiked in C3(MA). This result validates the use of our ELISA for measuring C3(H_2_O) in human specimens and ensures the absence of inhibitory matrix effects from serum at a 1:100 dilution (Figure 2D).

To further validate the ELISA, the inter- and intra- assay precision was evaluated. First, we determined the inter-assay variation, i.e., the variation between plates. Serum samples from 3 individuals were diluted 1:100 and 1:200 and assayed in triplicate on 3 separate plates. The inter-assay coefficient of variation (% CV) was calculated from the individual plate means (% CV 9.330; Table 1). Next, we determined the intra-assay precision, i.e., the variation between triplicates at 1:100 and 1:200 dilutions. The intra-assay % CV was calculated from 18 samples measured in triplicate and the average % CV was 5.849 (Table 2).

**Table 1:** Inter-assay precision.

	**1:100**			**1:200**		
	**P1**	**P2**	**P3**	**P1**	**P2**	**P3**
Mean of Means	4,547	3,210	2,441	4,785	3,524	2,648
Std Dev of Means	363.6	301.3	117.8	897.1	226.3	227.8
% CV of Means	7.997	9.384	4.826	18.75	6.422	8.602
Inter-assay CV (*n* = 3) = 9.330; 7.402 (1:100); 11.258 (1:200)						

*Mean levels expressed in ng/ml*.

**Table 2:** Intra-assay precision.

**Sample**	**Mean of triplicates**	**Standard deviation**	**% CV**
1	4,681	55.5	1.186
2	4,180	111.0	2.656
3	3,047	183.1	6.010
4	3,276	289.0	8.823
5	2,497	18.5	0.7410
6	2,520	293.4	11.64
7	4,824	181.6	3.766
8	4,360	127.1	2.914
9	3,026	5.132	0.1696
10	3,578	287.5	8.036
11	2,521	87.43	3.468
12	2,911	176.5	6.063
13	4,135	293.5	7.098
14	5,816	1,034	17.78
15	3,558	257.4	7.236
16	3,719	473.2	12.72
17	2,306	69.34	3.007
18	2,513	49.67	1.976
Intra-assay CV (n- = 18; 6/plate) = average % CV = 5.849			

*Mean levels expressed in ng/ml*.

Prior determinations of C3(H_2_O) in human samples have been confounded by the inability to specifically distinguish C3(H_2_O) from C3 and C3 activation products. Thus, one major goal of the C3(H_2_O)-specific ELISA was to obtain a more accurate measurement of human serum and plasma C3(H_2_O) levels. To demonstrate the utility of this assay for human samples, we established baseline C3(H_2_O) concentrations in human serum and plasma from healthy donors that were collected with different anticoagulants and analyzed immediately after collection (i.e. never frozen). We found that C3(H_2_O) levels in human sera, EDTA-plasma and heparin plasma were similar to one another (463.3 ± 51, 516.4 ± 52 ng/ml and 446.2 ± 51 ng/ml C3(H_2_O), respectively) (Figure 2E). In addition, to further confirm specificity of the assay and to determine how increased C3b/iC3b levels in samples might interfere with measurements of C3(H_2_O), we performed competition assays. We found that 50% inhibition of the 40 ng/ml C3(H_2_O) signal occurred at 5500 ng/ml of C3b and 5000 ng/ml iC3b (Figure 2F). The expected iC3b concentration in sera from an SLE patient is 5.22-5.95 μg/ml (27), so the background iC3b concentration at a 1:100 dilution typically used in this assay of ~50-60 ng/ml would have a negligible effect on the C3(H_2_O) measurement. Thus, interference by C3b or iC3b in serum is not likely to be a concern for accurate C3(H_2_O) measurements.

### Implications for Handling of Human Specimens

**Figure 3:**
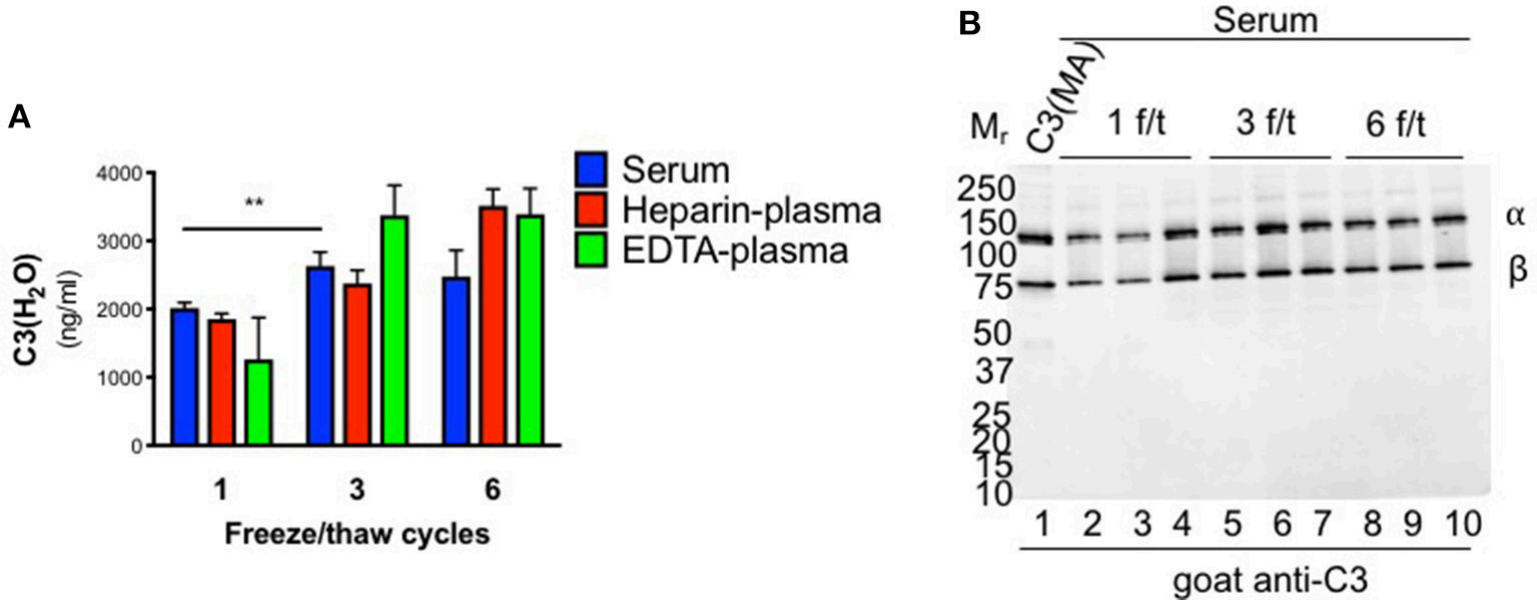
Effect of freeze/thaw cycles on C3(H_2_O) generation. **(A)** Samples were subjected to 1, 3 or 6 freeze/thaw (f/t) cycles and the C3(H_2_O) content was measured; *n* = 3, ^**^p < 0.01. **(B)** C3 cleavage was assessed by WB in serum subjected to increasing f/t cycles. No evidence of C3(H_2_O) degradation is observed. Notably, the sensitivity of the WB is ~10 ng and even an over-exposure fails to show additional bands.

Since sera are typically frozen prior to analysis, we also evaluated C3(H_2_O) levels in samples previously stored at −80°C. There was an approximately four-fold increase in C3(H_2_O) in serum following one freeze/thaw (f/t) cycle compared to freshly isolated (1,999 ± 111 vs. 463.3 ± 51; *p* < 0.0001; Figures 2E, 3A and Table 3). Consistent with this result, freezing and thawing of purified human C3 is reported to result in C3(H_2_O) generation and subsequent decrease of the hemolytic activity of complement (4). To more systematically evaluate this point in serum and plasma (vs. purified C3), we performed increasing numbers of f/t cycles on samples from healthy donors. Although there was as expected (see above), an increase in C3(H_2_O) content after a single f/t, subsequent f/t cycles had less of an impact on C3(H_2_O) generation, with a lesser, but significant, increase from 1 f/t to 3 f/t cycles and no difference after 6 f/t cycles (Figure 3A and Table 3). In contrast, we also evaluated generation of C3(H_2_O) from purified C3 and found that ~25% of the C3 had converted to C3(H_2_O) following 6 f/t cycles (Figure S3A), consistent with previous reports (4). Thus, C3 is stabilized against conversion to C3(H_2_O) in serum. These data indicate that interpretation of results must be adjusted for sample handling conditions (i.e., fresh vs. frozen serum or plasma).

**Table 3:** C3(H_2_O) levels in healthy controls (HC) and SLE patient sera.

	**Fresh serum**	**1 f/t**	**3 f/t**	**6 f/t**
Healthy control	463 (±51)	1,999 (±111)^****^	2,630 (±205)^π^	2,480 (±381)
SLE patient	ND	3,399 (±538)^**^	5,698 (±676)^#^	7,192 (±1090)^*^

Mean C3(H_2_O) level in ng/ml ± SE. ND = no data. F/T = Freeze/thaw. n = 3-6 (3 HC, 6 SLE);

**** p < 0.0001 vs. fresh serum;

** p < 0.01 vs. healthy control;

π p < 0.01 vs. healthy control 1 f/t;

# p < 0.0001 vs. SLE 1 f/t;

* *p < 0.05 vs. SLE 3 f/t*.

To evaluate the possibility that C3(H_2_O) was degraded (to iC3(H_2_O), for example), we ran WBs on the human sera that had undergone multiple f/t cycles. Intact C3 α- and β-chains were observed (Figure 3B), demonstrating that iC3(H_2_O) was indeed not being generated. Notably, at baseline, we do not detect the α_41_ fragment (~41 kDa) that is generated upon cleavage of C3(H_2_O) to iC3(H_2_O), indicating that, although our assay detects iC3(H_2_O), the majority of what we are detecting in serum samples is intact C3(H_2_O) (Figure 3B, lanes 2-4). From these data we concluded that the majority of C3(H_2_O) in serum samples, even when undergoing multiple f/t, is not in the form of iC3(H_2_O).

**Figure 4:**
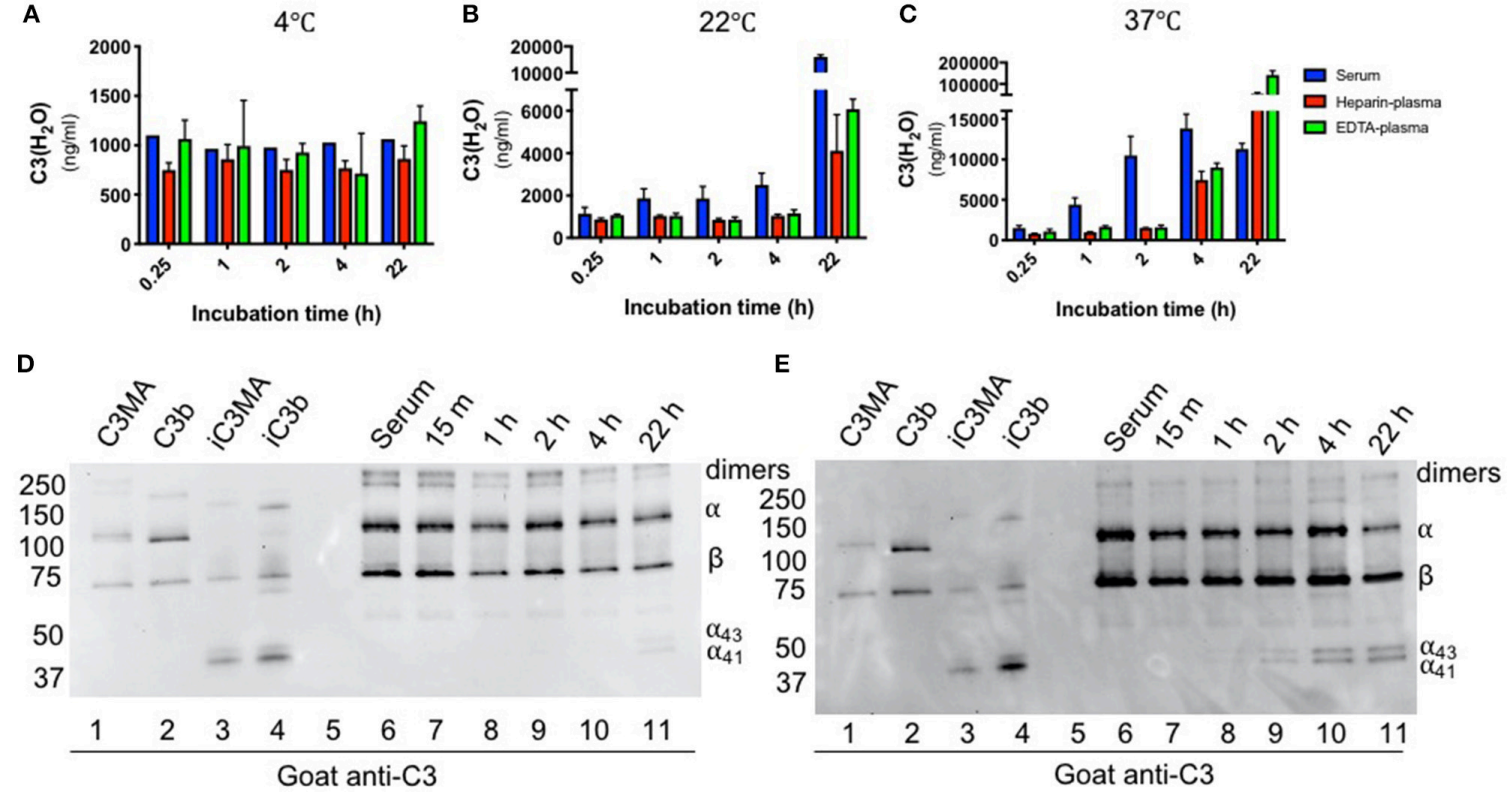
Effect of time and temperature on C3(H_2_O) generation in serum and plasma. Samples were incubated for the indicated time at 4°C **(A)**, 22°C **(B)** or 37°C **(C)** and then stored at −80°C prior to analysis; n = 3 healthy donors. C3(H_2_O) generation (i.e., tickover) does not occur up to 22 h at 4°C, occurs minimally over 4 h at RT, but occurs more rapidly (particularly in serum) at 37°C. Serum samples incubated at RT **(D)** or 37°C **(E)** were diluted 1:500 and assessed for C3 breakdown by WB under reducing conditions with a goat anti-C3 pAb. Note that we routinely detect low level contamination of C3 dimers in all purified C3 preps and sera samples that we evaluate by WB. Dimers are indicated in figure.

We also utilized the assay to examine C3(H_2_O) generation in healthy donors as this could facilitate a better understanding of how samples should be handled in the research laboratory and clinic. Our assay enabled us to revisit and quantitatively evaluate C3 activation in serum and plasma, which is thought to occur spontaneously and continuously at a low level (3–5). To evaluate the rate of C3(H_2_O) generation over time, healthy human donor serum and plasma samples were incubated *ex vivo* for up to 22 h at 4°C, room temperature (RT, 22°C) or 37°C. We did not detect C3(H_2_O) generation at 4°C, indicating that C3 in serum and plasma was stable at this temperature. At RT and 37°C, however, C3(H_2_O) was generated in both human serum and plasma (Figures 4A–C). Remarkably though, C3 is stable (minimal to no C3(H_2_O) generation) in EDTA- and heparin-plasma at 37°C for 2 h, demonstrating one advantage of plasma over serum. Notably, the level of C3(H_2_O) in serum incubated at 37°C is higher at 4 h compared to 24 h. We hypothesize that this decrease is due to cleavage of the C3(H_2_O) to fragments that are not detected by our ELISA. This hypothesis is supported by the reduction in the α-chain of C3 on WB at this timepoint (Figure 4E, lane 11). Importantly, though, our results suggest that serum samples may be stored at 4°C for up to at least 22 h without C3(H_2_O) generation.

Although we did not detect significant C3(H_2_O) generation after 1 h at RT in undiluted serum (above), we evaluated generation of C3(H_2_O) in diluted serum during the capture step of the ELISA to confirm that this incubation step is not altering C3(H_2_O) measurements. This was accomplished by incubating a 1:100 dilution of NHS over a time course and then determining C3(H_2_O) concentration using a standard curve captured for the equivalent amount of time. Standard curves captured for the corresponding time was important to ensure that binding time was not the reason for differences in C3(H_2_O) measurements. In 1:100 NHS, we did not detect a significant increase in C3(H_2_O) generation over the 1 h capture time at RT, indicating that samples are stable under these assay conditions (Figure S3B).

The increase in C3(H_2_O) at elevated temperatures may also drive accelerated production of C3 activation products. Using WB, we found that the kinetics of iC3b/iC3(H_2_O) (i.e presence of C3 α_43_ and α_41_ fragments) appearance were similar to that of C3(H_2_O) generation, beginning at 22 h at RT (Figure 4D) and 2 h at 37°C (b). This suggests that, like its generation, inactivation of C3(H_2_O) by Factors H and I occurs more rapidly at 37°C. However, we do not detect the α_24_ band, even upon overexposure of the WB, indicating that there is no further cleavage to C3c (Figure S4). Since our ELISA also detects iC3(H_2_O), there is no evidence that breakdown affected our detection of C3(H_2_O) levels. Taken together, these studies demonstrate the effectiveness of our assay using human samples and provides guidance into how human specimens should be handled and stored for C3(H_2_O) and overall complement measurements (see section Discussion).

### C3(H_2_O) Levels Are Increased in SLE and RA Sera

**Figure 5:**
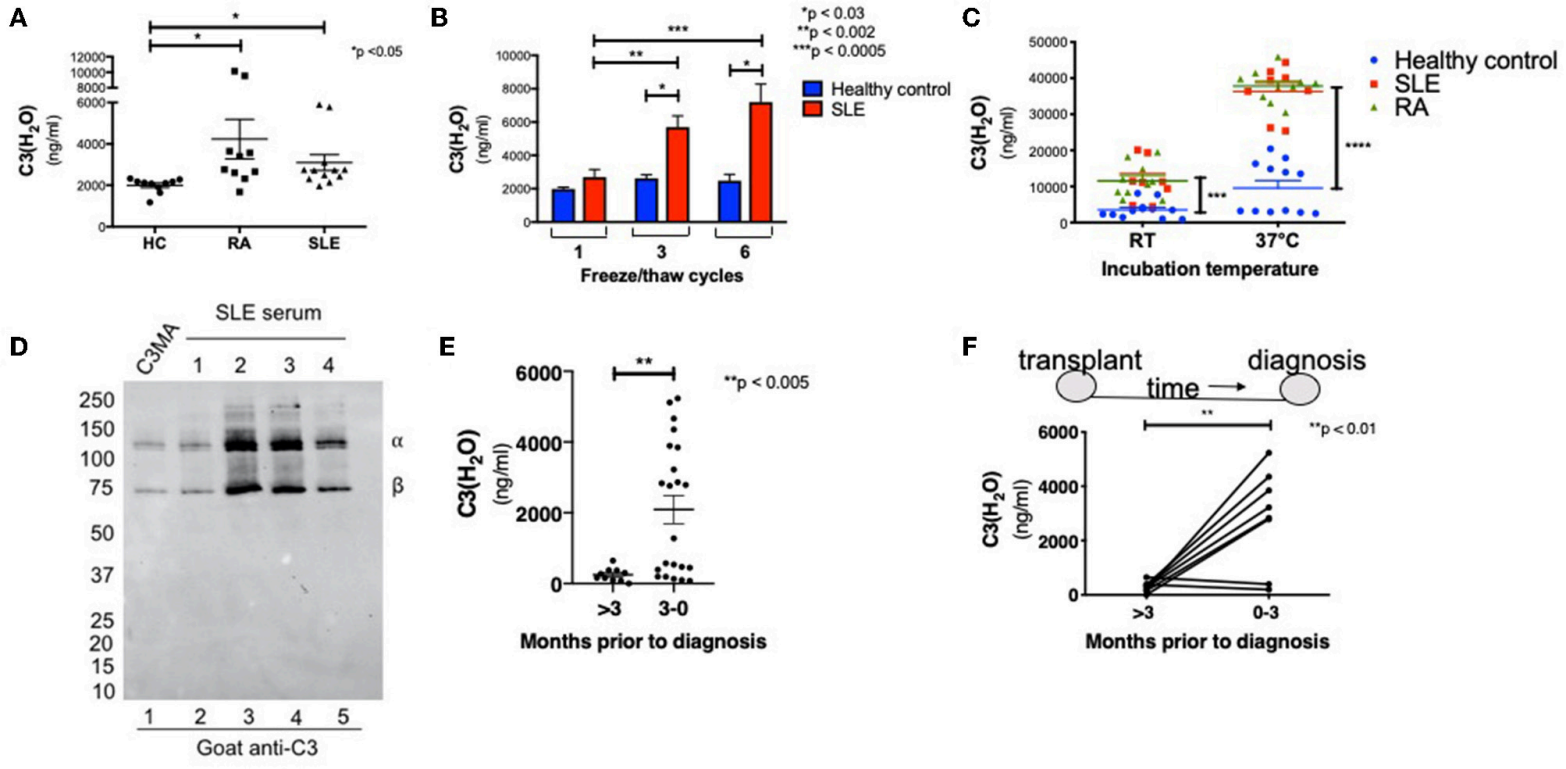
C3(H_2_O) is elevated in complement-mediated conditions. **(A)** C3(H_2_O) levels were measured in serum samples from RA and SLE patients and compared to serum from healthy donors. All samples had been stored at −80°C prior to evaluation; n = 5 (in duplicate; intra-assay precision, %CV 5.59). **(B)** Serum from SLE patients were subjected to 1, 3, or 6 f/t cycles prior to measuring C3(H_2_O) levels; *n* = 3. **(C)** Serum from healthy donors (*n* = 6) or patients with SLE (*n* = 4) or RA (n = 5) were incubated at RT or 37°C for 6 h before C3(H_2_O) was measured; samples in duplicate. SLE at 22°C, ^***^p < 0.0005 vs. healthy control; RA at 22°C, SLE and RA at 37°C, ^****^p < 0.0001 vs. healthy control. **(D)** Serum from SLE patients (*n* = 4) was diluted 1:500 and assessed for C3(H2O) breakdown by WB under reducing conditions with a goat anti-C3 pAb. **(E)** C3(H_2_O) levels were measured in BAL fluid samples from lung transplant recipients either >3 months prior to diagnosis (*n* = 10) or within 3 months of diagnosis of AMR (*n* = 22), ^**^p < 0.005 by student's *t-*test. **(F)** C3(H_2_O) levels were measured in sequential BAL fluid samples in 8 patients who had paired specimens. Lines connect sequential samples from the same patient; ^**^p < 0.01 by paired *t*-test. HC, healthy control; SLE, systemic lupus erythematous; RA, rheumatoid arthritis.

Having established the specificity, sensitivity and sample handling conditions for the C3(H_2_O) ELISA, we next desired to evaluate the diagnostic utility of this assay. Thus, levels of C3(H_2_O) were measured in serum from patients with autoantibody-associated diseases where complement activation is known to occur (38–40). These samples and the corresponding healthy donor samples had all been stored at −80°C prior to analysis. We found an increase in C3(H_2_O) levels in patients with active SLE (3,098 ± 382 ng/ml) and RA (4,234 ± 955 ng/ml) compared to healthy controls (HC; 1,999 ± 111 ng/ml) (Figure 5A), suggesting modestly higher baseline levels in patients' serum in these two disease states.

Since we did not detect significant increases in C3(H_2_O) generation in healthy donors after the initial f/t cycle (Figure 3A), we hypothesized that this would also be true utilizing autoimmune patient samples. Instead, we found that C3(H_2_O) increased in serum samples from SLE patients with each f/t cycle (Figure 5B and Table 3), establishing that the rates of C3(H_2_O) generation are increased in SLE sera. To confirm this observation, we incubated sera from healthy control, RA and SLE patients *ex vivo* at RT or 37°C for 6 h. The difference in C3(H_2_O) levels following incubation was much greater than the baseline differences, with an accelerated rate of C3(H_2_O) generation in SLE and RA patients (Figure 5C). This indicates that the potential for C3 turnover is higher in SLE and RA patients compared to healthy donors. Since there is a higher concentration of C3(H_2_O) in SLE serum compared to healthy controls, we confirmed by WB that in SLE samples the majority is in the form of C3(H_2_O), not iC3(H_2_O) (Figure 5D). These data support the possibility that C3(H_2_O) may serve as a biomarker in SLE and RA.

### C3(H_2_O) Levels in BAL Fluid From Lung Transplant Recipients

We also wanted to determine whether increased C3(H_2_O) levels could be observed in other complement-mediated conditions featuring local, rather than systemic, complement activation. Thus, we examined C3(H_2_O) levels in bronchoalveolar lavage (BAL) fluid samples obtained from lung transplant recipients either more than 3 months prior to antibody-mediated rejection (AMR) diagnosis or within 3 months of this diagnosis. We found a significant increase in C3(H_2_O) in the BAL fluid obtained within 3 months prior to AMR compared to BAL samples obtained further from diagnosis (Figure 5E; 2,091 ± 398 vs. 248.3 ± 60 ng/ml; *p* < 0.005). Additionally, we evaluated sequential BAL specimens from lung transplant recipients who developed AMR to compare C3(H_2_O) changes in individual patients. In 6 of 8 patients, C3(H_2_O) level increased in the BAL fluid within 3 months prior to AMR diagnosis (Figure 5F). These data strongly support the potential utility of clinically evaluating C3(H_2_O) levels in the BAL as a both a diagnostic and, more importantly, a putative prognostic biomarker of AMR. They further demonstrate the potential utility of the assay to quantify C3(H_2_O) in human fluids beyond serum and plasma.

## Discussion

C3(H_2_O), described nearly 40 years ago as the key form of C3 triggering alternative pathway activation (3–5, 41), has remained somewhat of an enigma as to how it is generated *in vivo* and, particularly, its potential importance in a clinical setting. Also, the recent discoveries of an intracellular complement system (11) and a C3(H_2_O) cellular recycling pathway (20) have reinvigorated interest in this distinct form of C3, especially relative to defining its utility as a biomarker and initiator of complement activation. While clinical assays exist for C3 and certain of its fragments (C3a, iC3b and C3d), currently there is no sensitive and straight-forward assay available to measure C3(H_2_O). In this report, we provide such an assay and further hypothesize that C3(H_2_O) is a previously underappreciated player in autoimmunity and inflammatory diseases whose measurement may serve as a biomarker. In this manuscript, utilizing a specific and sensitive assay for C3(H_2_O), we provide: 1) data on which one can form guidelines for handling of human biospecimens for complement measurements; 2) an analysis of the effects of temperature and freeze-thawing on C3(H_2_O) generation in serum and plasma samples; and 3) three examples of diseases in which C3(H_2_O) measurements may inform on disease pathogenesis and/or serve as a diagnostic or prognostic biomarker.

In fresh serum and heparinized- or EDTA-treated plasma samples from healthy donors, the concentration of C3(H_2_O) was ~500 ng/ml, a value lower than the 10-20 μg/ml that would have been predicted from previous reports demonstrating 1–2% of purified C3 turning over per hour (4, 5). There was no statistical difference in C3(H_2_O) levels between serum or the two types of plasma. However, if either the serum or plasma samples were frozen once the C3(H_2_O) levels increased 3- to 4-fold. Further, in contrast to our expectations, subsequent freeze-thaw cycles (up to 6x) had a lesser influence on the C3(H_2_O) concentration: thus, it was not proportionally generated by repetitively freezing and thawing of the samples which may have been expected from the literature (4). The effect of temperature was also studied. Notably, maintaining the sample at 4°C led to no further generation of C3(H_2_O) for up to 22 hrs. In contrast, at 37°C or 22°C C3(H_2_O) levels began to increase at 2 or 4 h of storage, respectively.

One explanation for the apparent discrepancy in C3(H_2_O) levels between this study and previous work is that we have serum or plasma instead of purified C3 in aqueous solution. In our assay system, we demonstrate a rather dramatic (~100- fold) difference in C3(H_2_O) formation by purified C3 in aqueous buffer vs. C3 in plasma or serum following freeze-thaw cycles (Figure S3A). Purified C3 (in the presence of factors B, D, H, and I) is reported to be inactivated at a rate of 1–2%/h at 37°C (4, 5). In the absence of additional serum factors (i.e., purified C3 alone in buffer), the generation of C3(H_2_O) was reported to occur at 0.2–0.4%/h (4, 5). In line with these previous observations, C3(H_2_O) was generated in serum or plasma incubated at 37°C at ~0.3–0.5%/h, although this was not linear at longer time points. We hypothesize that the lack of linearity is due to either regulation by FH at higher concentrations of C3(H_2_O) or degradation by other proteases. This is consistent with the previous reports showing C3 inactivation at 1–2%/h in the presence of additional serum factors (3, 4). Additionally, the binding site for FH in C3(H_2_O) is not exposed immediately due to the slow conformation change of this protein (7) and this may explain the observation of delayed regulation of the C3(H_2_O) by FH in our system. A more comprehensive understanding of C3(H_2_O) regulation is an active focus of our laboratory.

One goal of our study was to better understand and thereby provide further guidance as to how human samples should be handled in the research laboratory and clinic for complement measurements. We note that previous work suggests that C3(H_2_O) generation occurs due to contact activation [reviewed in (42)] and therefore incubating serum and plasma in tubes, as we have in our experiments, does not reflect *in vivo* generation of C3(H_2_O). However, human biospecimens are stored this way both in the laboratory and in the clinic so our data are instructive relative to the handling and storage of samples and the interpretation of data from patient-derived specimens, where complement activation and turnover may be playing a role. Specifically, our results provide guidelines for a suitable short-term storage temperature for human serum or plasma prior to clinical testing or investigative experiments in the laboratory setting.

Our data also support the assessment of C3(H_2_O) generation as a biomarker for the presence of autoantibody-mediated diseases. Identifying such a biomarker that may be tied to the underlying pathogenesis would also fill a significant unmet need in the evaluation of patients with autoimmune diseases. Since C3 levels in blood are altered in SLE, it may be more instructive to express the C3(H_2_O) level as a ratio of total C3 (27). Regardless, C3(H_2_O) generation is enhanced in SLE and RA patients and we will explore in detail the reasons in our patient cohorts in the future.

The observed differences in C3(H_2_O) generation may represent a fundamental variation in C3 metabolism in SLE and other diseases featuring complement activation. The two major consequences of a complement system deficiency are bacterial infections and autoimmunity (particularly SLE) (38, 43). In acute injury states leading to membrane damage, apoptosis and necrosis, the complement system plays an important role in the host's response to “altered self” (44). The function of C3(H_2_O) in these processes is unclear and has not been specifically investigated. However, the discovery of an intracellular complement system (11), including the observations that C3(H_2_O) uptake is a major source of intracellular C3 (20) that modulates key cytokines (17) and promotes cell survival (45), may provide a better understanding of etiopathology of certain inflammatory disease and lead to more effective treatment options.

Although the mechanism of accelerated C3(H_2_O) generation in SLE and RA is unknown, we hypothesize that augmented inherent instability in the patients' C3 protein driven by the presence of a proinflammatory environment and damaged or injured cellular tissues is at the basis of pathogenesis. To that end, our assay will allow quantification of C3(H_2_O) in both *in vitro* and *in vivo* systems to better understand its role and mechanism of generation. Using our SLE cohort (27), we plan to study C3(H_2_O) in those patients with low complement, active vs. inactivate disease, and varying severity of organ involvement. Our goal is to assess these and multiple other aspects relative to the requirements *in vivo* for C3(H_2_O) generation. We will utilize this assay to better understand how complement activation drives diseases pathogenesis and severity and whether it can be used as a diagnostic biomarker. Additionally, these data do not rule out that C3(H_2_O) may also be a marker of disease activity in SLE and we will explore this and other possibilities in our SLE patient cohort (27) in the future.

We also wanted to determine whether increased C3(H_2_O) levels could be observed in other complement-mediated conditions featuring local, rather than systemic, complement activation. Acute antibody-mediated rejection (AMR) is a rare and often fatal complication that occurs following lung transplantation (46, 47). AMR is associated with objective measurements of allograft dysfunction (decline in lung function, infiltrates on x-rays, histopathological changes on biopsy), which can present with or without symptoms. AMR is defined as allograft dysfunction in the presence of donor-specific antibodies to HLA (DSA) along with positive histology suggestive of AMR (such as neutrophil margination, capillaritis, organizing pneumonia, and /or lung injury) and positive C4d staining (47). However, not all patients present as “definite clinical” AMR; some may lack all of these features, and in some, C4d staining can be negative (48). Moreover, while the presence of DSA in asymptomatic patients is a risk factor for AMR, there is no predictive biomarker for this condition, especially one that can be obtained without an invasive transbronchial biopsy (47).

Our results suggest that C3(H_2_O) increases in the BAL fluid of lung transplant recipients prior to the development AMR. While complement activation has been connected with AMR (46, 47, 49), C4d staining in lung of AMR patients is neither sensitive nor specific, and an entity of C4d-negative AMR is also increasingly appreciated (48). Moreover, C4d staining needs a biopsy to be performed, and is most likely to be positive around the time of AMR diagnosis. Hence, further evaluating a biomarker that does not need a biopsy to be performed should be of interest to the transplant community. In future studies, our assay can be used to determine if these C4d-negative cases are truly mediated by a pathological process that is independent of complement activation; or whether C3(H_2_O) would be a more specific and non-invasive biomarker compared to C4d staining (50, 51). Thus, our results prompt not only the potential use of C3(H_2_O) as a biomarker for AMR but also investigating C3(H_2_O) development and function to better understand mechanisms driving AMR.

As our study points out, it is critical to understand proper sample handling and to utilize the appropriate comparator (i.e., frozen vs. fresh healthy control serum) when interpreting the C3(H_2_O) content of patient samples. Additionally, studies by Pangburn et al (37) have demonstrated the presence of a C3(H_2_O) intermediate, designated C3(N)^*^, that has not adopted the C3b-like final conformational state of C3(H_2_O) (25, 37). This intermediate would not be detected by our ELISA, since it recognizes only the final conformational state C3(H_2_O). We plan to better understand the potential impact of C3(N)^*^ on the interpretation of the results of the C3(H_2_O) assay in human biospecimens. For example, whether this C3(N)^*^ is one of the reasons for the 3 to 4x increase in C3(H_2_O) associated with one freeze-thaw or the increased generation observed in SLE and RA samples remains to be investigated. The functional role and relevance of this intermediate to human disease is also under investigation.

While an ELISA has been previously described to quantitate C3(H_2_O), the mAb utilized had higher specificity to C3a (21, 22). This was an inherent limitation for the assay to specifically quantify C3(H_2_O), as the presence of C3a in a sample would interfere with the detection of C3(H_2_O) in this ELISA. In the ELISA described herein, we detect only C3(H_2_O) and its cleavage fragment iC3(H_2_O) and are able to exclude the effects of C3a, C3b, iC3b, C3c, C3dg, or C3d on measurements. The additional C3(H_2_O) cleavage fragment that we do measure, iC3(H_2_O), would be generated by cofactor activity. However, when we assessed this possibility, the large majority of protein we detected in serum and plasma from healthy controls and SLE patients was C3(H_2_O), not its degradation product iC3(H_2_O). Thus, this C3(H_2_O) ELISA provides an opportunity to investigate the role of C3(H_2_O) in mediating complement activity in human health and disease.

In summary, we have developed and characterized an ELISA that is specific for human C3(H_2_O). The role of C3(H_2_O) in autoimmunity and inflammation has not been thoroughly investigated in part because of difficulty of measuring it quantitatively in human fluids. The C3(H_2_O) ELISA represents a tool to allow for further analysis of the role of C3(H_2_O) in human autoimmune and inflammatory diseases. It also could assist in the identification of intracellular pathways of complement activation that would disclose novel therapeutic targets. Notably, the commercial availability of the antibodies required to set-up this ELISA make it easily adoptable by any interested lab. Additionally, our data using the C3(H_2_O) ELISA suggests that C3(H_2_O) in the plasma should be further rigorously evaluated as a promising biomarker for SLE and in the lungs, as a prognostic indicator in lung transplant recipients at an increased risk for developing acute antibody-mediated rejection.

## Data Availability

All datasets generated for this study are included in the manuscript and/or the Supplementary Files.

## Author Contributions

JA, ME, ML, HK, and AK designed the research studies and analyzed and interpreted the data. ME, ML, and AL conducted experiments and acquired data. AK, HK, RH, and TM provided patient samples. ME drafted the manuscript, and all authors contributed revisions.

### Conflict of Interest Statement

The authors declare that the research was conducted in the absence of any commercial or financial relationships that could be construed as a potential conflict of interest.

## Abbreviations

C3(H_2_O): hydrolyzed form of C3
C3(MA): C3 treated with methylamine to hydrolyze the thioester
iC3(H_2_O), C3(H_2_O) cleaved by factor I in the presence of factor H, iC3(MA): C3(MA) cleaved by factor I in the presence of factor H
f/t: freeze-thaw
pAb: polyclonal antibody
WB: Western blot
FB: factor B
FI: factor I
FH: factor H
RT: room temperature
ICS: intracellular complement system
SLE: systemic lupus erythematosus
RA: rheumatoid arthritis
AMR: antibody mediated rejection
BAL: bronchoalveolar lavage
NHS: normal human serum.
