# Supplementary material for: Development and Optimization of an ELISA to Quantitate C3(H2O) as a Marker of Human Disease
Source: Front Immunol. 2019 Apr 4;10:703. doi: 10.3389/fimmu.2019.00703 (PMC6458276; doi:10.3389/fimmu.2019.00703)
Supplement: Supplementary file 1 [file Data_Sheet_1.PDF]

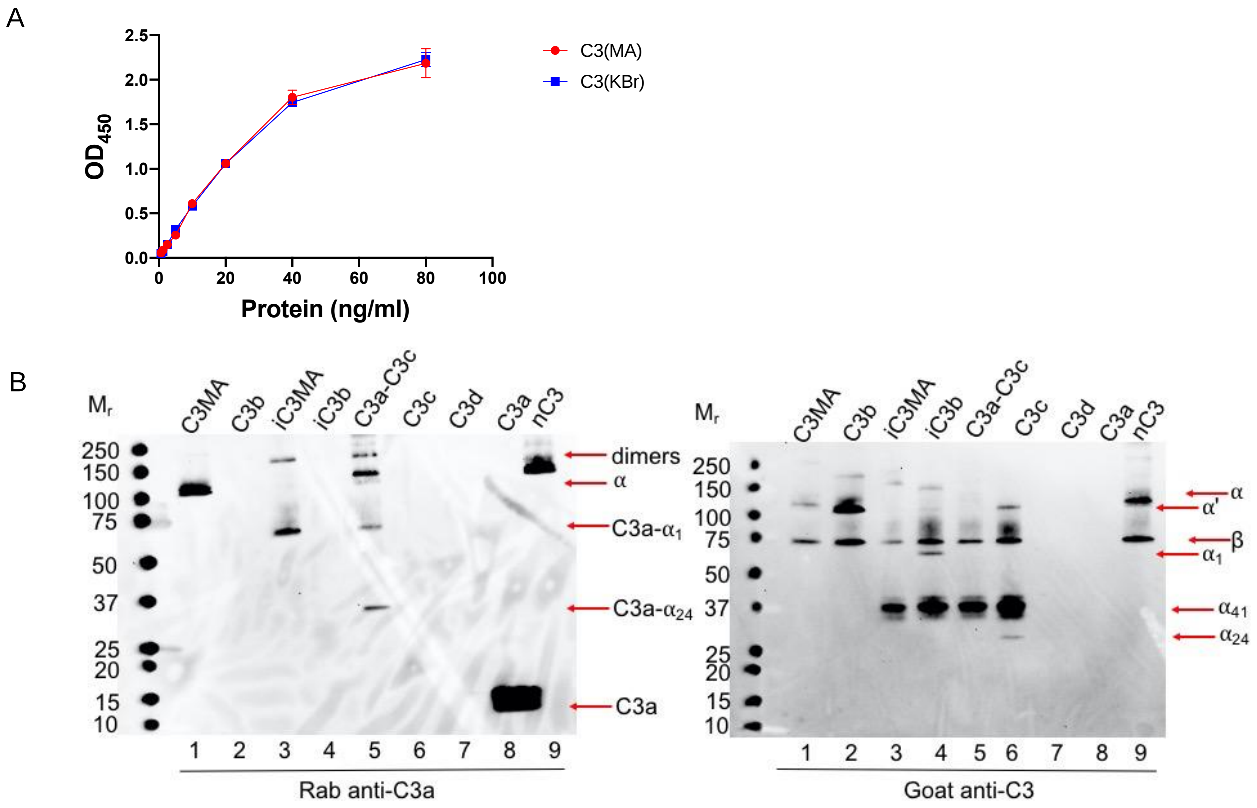

Figure S1. Specificity of $C3(H_2O)$ ELISA. C3(KBr) and C3(MA) are recognized comparably in the $C3(H_2O)$ ELISA **(A).** Increasing concentrations (1-80 ng/ml) of C3(MA) and C3(KBr) were compared in the $C3(H_2O)$ ELISA. n=4, representative of 2 independent experiments. **(B)** Following treatment with FH or CR1 and FI, the proteins were assessed by WB under reducing conditions.

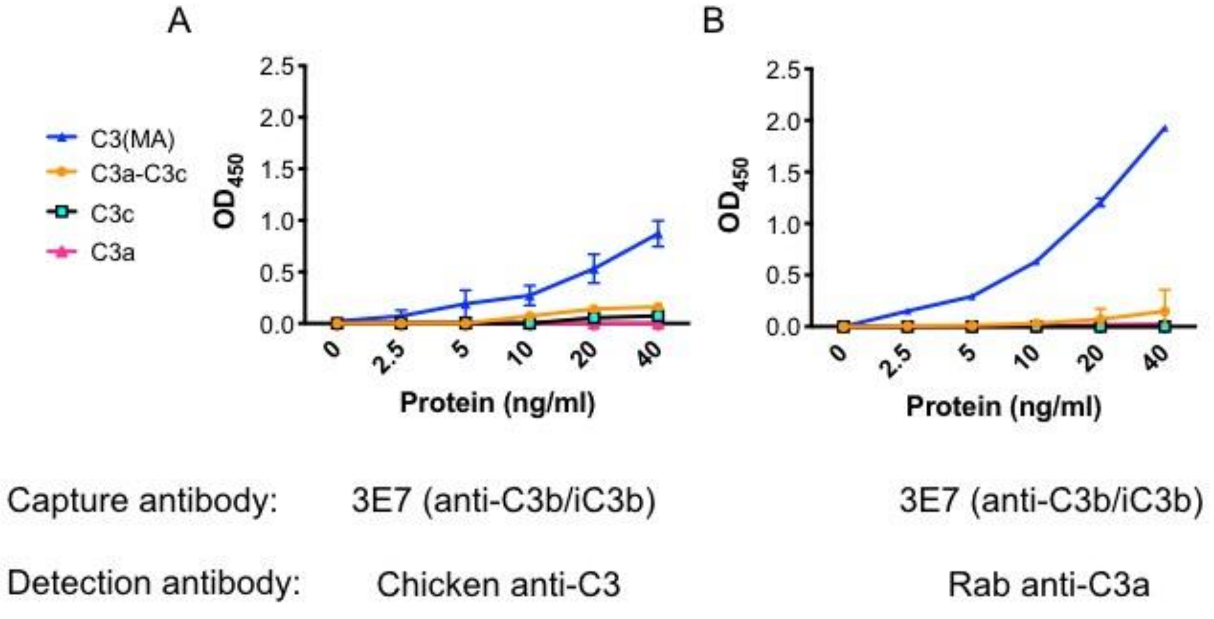

Figure S2. Specificity of the C3(H_2O) ELISA. (A) Binding comparison of proteins using anti-C3 pAb for detection**. (B)** Specificity of ELISA employing anti-C3a pAb. C3a-C3c, C3c and C3a are not detected.

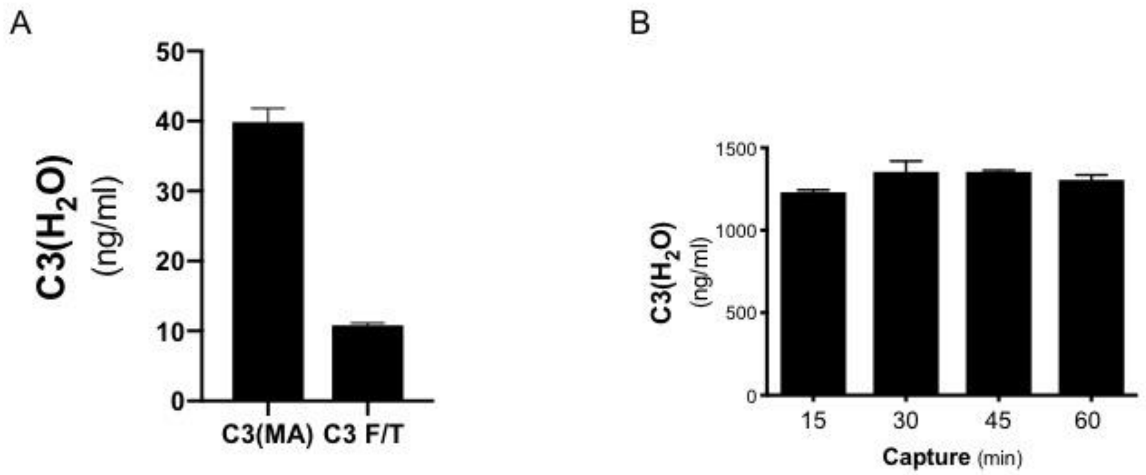

Figure S3. ELISA optimization. (A) Purified C3 (1 mg/ml) was subjected to 6 f/t cycles (C3 F/T). The C3(H_2O) content of C3 F/T was determined by loading 40 ng/ml of the protein and standard [C3(MA)] in the ELISA. Following 6 f/t cycles 25% of the purified C3 preparation has converted to C3(H_2O) [i.e. ~10 ng of the 40 ng C3 F/T loaded is C3(H_2O)], thus approximately 250 μg/ml of the undiluted 1 mg/ml C3 F/T preparation is C3(H_2O). n=2. **(B)** Generation of C3(H_2O) in 1:100 dilution of NHS over a 1 h capture incubation step of the ELISA was evaluated by measuring C3(H_2O) content by ELISA every 15 min during the capture step; n=2.

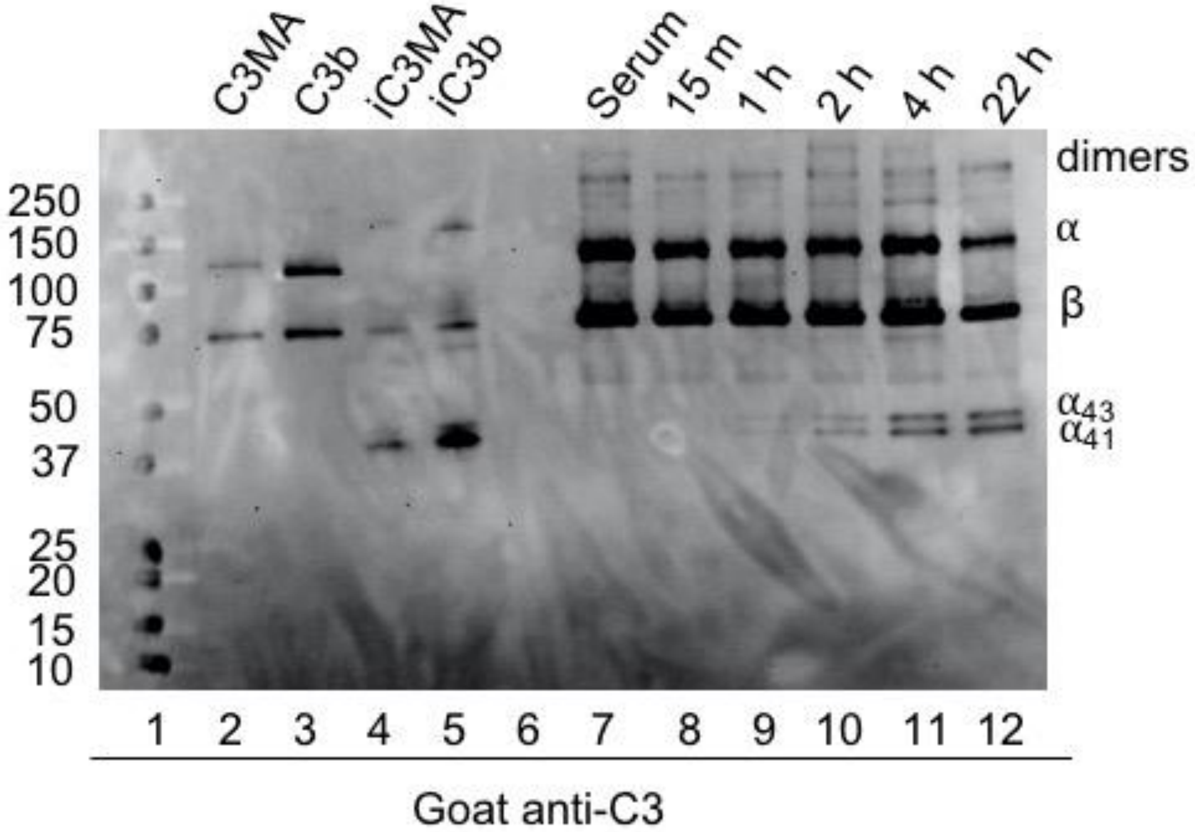

Figure S4. C3c generation is not detected after 22 h at 37°C. Uncropped and overexposed image of the WB shown in Figure 4E, demonstrating the absence of the α_{24} band that is diagnostic of C3c.
